# Hypokalemic Quadriparesis as the Initial Presentation of Sjögren's Syndrome With Distal Renal Tubular Acidosis: A Case Report

**DOI:** 10.7759/cureus.96825

**Published:** 2025-11-14

**Authors:** Sri Hari Babu Sunkari, Mrunali U Nikhare, Shoaib Baig, Jashwini J Bhoyar

**Affiliations:** 1 Department of Trauma and Emergency, All India Institute of Medical Sciences, Nagpur, Nagpur, IND

**Keywords:** distal renal tubular acidosis, hypokalemic quadriparesis, non-anion gap metabolic acidosis, severe hypokalemia, sjögren's syndrome

## Abstract

A 44-year-old woman with long-standing hypothyroidism presented with sudden-onset flaccid quadriparesis preceded by distal limb paresthesia, progressing rapidly to respiratory compromise requiring intubation. Laboratory evaluation revealed severe hypokalemia with non-anion gap metabolic acidosis, elevated transtubular potassium gradient, and inappropriately alkaline urine, consistent with distal renal tubular acidosis (dRTA). In the emergency, correction of electrolytes, intravenous bicarbonate therapy, and supportive management led to rapid neurological improvement. Following stabilization, further evaluation revealed positive antinuclear antibody (ANA) and anti-Ro/SSA, anti-La/SSB, and Ro52 antibodies, confirming Sjögren's syndrome. The patient had no prior sicca symptoms. This case highlights that hypokalemic paralysis can be the initial presentation of Sjögren’s syndrome through dRTA, preceding classical exocrine features. Early recognition and correction of metabolic derangements are crucial for preventing morbidity and facilitating the diagnosis of underlying autoimmune pathology.

## Introduction

Sjögren's syndrome (SS) is a systemic autoimmune disorder characterized by the lymphocytic infiltration of salivary and lacrimal glands, leading to xerostomia and keratoconjunctivitis sicca. Extraglandular involvement occurs in up to one-third of patients and may affect the kidneys, lungs, nervous system, and vasculature [1]. Among renal manifestations, distal renal tubular acidosis (dRTA) is the most frequent and may present with hypokalemia severe enough to cause flaccid paralysis [2]. Early recognition is crucial in the emergency department, as the timely correction of metabolic abnormalities can be lifesaving and may lead to the diagnosis of an underlying autoimmune disease [3]. We present the case of a 44-year-old woman who presented with acute flaccid quadriparesis due to hypokalemia secondary to dRTA as the first manifestation of SS.

## Case presentation

A 44-year-old woman with a known history of hypothyroidism for the past 10 years, maintained on thyroxine 25 µg daily, presented with acute-onset weakness involving all four limbs. The weakness was preceded by tingling and numbness that progressed rapidly over a few hours to complete inability to move. There was no history of fever, diarrhea, vomiting, trauma, recent vaccination, or toxin exposure. She denied prior similar episodes, and no family history of paralysis was noted. There were no symptoms suggestive of hyperthyroidism, bulbar weakness, sensory loss, or bladder and bowel involvement. At a peripheral hospital, she developed respiratory distress and was intubated for impending respiratory failure. Initial investigations there revealed severe hypokalemia with a serum potassium of 1.9 mmol/L. Nerve conduction studies performed at the peripheral hospital demonstrated reduced compound muscle action potential (CMAP) amplitudes in both upper and lower limb motor nerves, with preserved conduction velocities and nonrecordable F-wave latencies, raising concern for an acute motor axonal neuropathy (AMAN)-like pattern (Table 1). She was referred to our tertiary center for further management.

**Table 1:** Nerve conduction study showing reduced CMAP amplitudes and NR F-wave latencies interpreted as acute motor axonal neuropathy at the referring center "↓" indicates reduced amplitude compared to reference values. Only right-sided motor nerves are presented for the sake of convenience, as nerve conduction findings were symmetrical bilaterally. ms: millisecond; mV: millivolt; ms: meter/second; NR: nonrecordable; CMAP: compound muscle action potential

Nerve (motor)	Distal latency (ms)	Reference range	CMAP amplitude (mV)	Reference range	Conduction velocity (m/s)	Reference range	F-wave latency (ms)	Reference range
Median (right)	3.2	≤4.4	1.8 ↓	≥4.0	52	≥50	NR	≤32
Ulnar (right)	2.8	≤3.3	1.5 ↓	≥6.0	55	≥53	NR	≤33
Peroneal (right)	3.6	≤6.5	0.9 ↓	≥2.0	48	≥40	NR	≤56
Tibial (right)	4.0	≤6.0	1.1 ↓	≥4.0	47	≥41	NR	≤58

On arrival at our emergency department, the patient was conscious, oriented, and ventilated on controlled mechanical ventilation. Her pulse rate was 100 beats per minute, blood pressure 117/90 mmHg, and oxygen saturation 97% with 40% fraction of inspired oxygen (FiO2). Neurological examination showed hypotonia and symmetrical flaccid weakness involving both upper and lower limbs, with power graded as 2/5 in both the upper and lower limbs. Deep tendon reflexes were diminished in the upper limbs and absent in the lower limbs, with mute plantar responses bilaterally. Sensory examination was normal, and cranial nerve examination showed no deficits. Initial laboratory investigations are summarized in Table 2.

**Table 2:** Laboratory investigations at presentation

Parameter	Result	Reference range	Interpretation
Serum potassium	1.9 mmol/L	3.5-5.5 mmol/L	Severe hypokalemia
Serum sodium	135 mmol/L	135-145 mmol/L	Normal
Serum bicarbonate	14 mmol/L	22-26 mmol/L	Low - metabolic acidosis
Serum chloride	110 mmol/L	98-106 mmol/L	Hyperchloremia
Anion gap	11	8-12	Normal anion gap metabolic acidosis
Transtubular potassium gradient	~9	<3	Renal potassium wasting
Urine pH	6.0	<5.5	Alkaline

Following the diagnosis of dRTA, the patient was started on intravenous potassium chloride and supportive therapy. After initial management, differentials such as Guillain-Barré syndrome (GBS), thyrotoxic periodic paralysis, familial hypokalemic periodic paralysis, and myxedema-related paralysis were considered (Table 3). AMAN-variant GBS was initially considered due to reduced CMAP amplitudes, but the dramatic recovery within hours of potassium correction is not compatible with GBS. Severe hypokalemia can transiently mimic axonal neuropathy on nerve conduction study (NCS), which is sometimes initially misinterpreted as GBS [4]. Thyrotoxic paralysis was considered less likely due to the lack of hyperthyroid features. Familial periodic paralysis was improbable given the late age of onset and accompanying metabolic acidosis. Myxedema-related weakness was ruled out as the patient was clinically euthyroid. 

**Table 3:** Differential diagnoses for acute flaccid quadriparesis dRTA: distal renal tubular acidosis; GBS: Guillain-Barré syndrome

Condition	Key clinical features	Findings in the present case
GBS	Ascending paralysis, areflexia, albuminocytologic dissociation	Rapid improvement with potassium correction excludes GBS
Thyrotoxic periodic paralysis	Hypokalemia, hyperthyroidism	Patient euthyroid on treatment
Familial hypokalemic periodic paralysis	Young age, normal acid-base	Adult onset, metabolic acidosis - excluded
Myxedema-related paralysis	Untreated hypothyroidism, bradycardia, hypothermia	Absent - excluded
dRTA	Hypokalemia, metabolic acidosis, renal K⁺ wasting	Confirmed diagnosis

Within 12 hours, she showed marked neurological improvement, regaining partial motor strength, and was shifted to the ICU for close monitoring. Spine imaging was not considered in this case, as the metabolic etiology was evident and the patient demonstrated rapid neurological recovery following the correction of hypokalemia; however, spine imaging may be considered in similar presentations to exclude alternative neurological causes.

By 24 hours, the patient achieved full neurological recovery and was successfully extubated. She was then shifted to the ward for further evaluation. Further evaluation showed positive antinuclear antibody (ANA) (1:100, speckled pattern) and positivity for anti-Ro/SSA, anti-La/SSB, and Ro52 antibodies. Thyroid function was normal (thyroid-stimulating hormone (TSH) 3.5 µIU/mL). Schirmer's test and nailfold capillaroscopy were within normal limits. The patient was discharged on oral potassium citrate, hydroxychloroquine, and optimized thyroxine (50 µg/day) with advice for long-term rheumatologic follow-up. A chronological summary of clinical events is shown below (Table 4).

**Table 4:** Chronological summary of clinical events ANA: antinuclear antibody

Time since presentation	Event
Before arrival at our hospital	Sudden onset of weakness; intubation for respiratory distress at another hospital
At the time of presentation	Severe hypokalemia and metabolic acidosis confirmed
0-6 hours	Potassium and bicarbonate correction initiated; neurological recovery started
12-24 hours	Complete neurological recovery; patient extubated
Days 2-3	Autoimmune testing positive for ANA and anti-Ro/La; diagnosis of Sjögren's syndrome confirmed
Day 4	Initiated on hydroxychloroquine and oral potassium citrate
Day 5	Discharged stable; potassium 4.1 mmol/L

## Discussion

Hypokalemic paralysis due to dRTA in SS is a rare but well-recognized association. In SS, autoimmune-mediated lymphocytic infiltration of the renal interstitium impairs distal tubular H⁺ secretion, resulting in hyperchloremic metabolic acidosis, alkaline urine, and renal potassium loss [5,6]. Our case exemplifies the uncommon scenario where paralysis preceded any exocrine gland symptoms, highlighting that renal and neurological manifestations can occur early. dRTA is the most frequent renal manifestation of SS, occurring in up to 33% of cases, but clinically significant hypokalemia appears in fewer than 5% [7,8]. Similar presentations have been described by Meena et al. [3], Yadav et al. [9], Asmara et al. [10], and Sedhain et al. [11], where hypokalemic paralysis was the first clue to underlying SS. Ravisankar et al. [6] also reported a similar case where such a presentation led to the diagnosis of primary SS. The combination of metabolic acidosis and muscle paralysis may mimic GBS or periodic paralysis, potentially delaying recognition [12]. Early correction of potassium and acidosis rapidly reverses neuromuscular dysfunction, as seen in our patient, whereas untreated cases may progress to respiratory failure or arrhythmia [2,7].

Compared to previously published reports, Sarah et al. [7], Das et al. [8], Hamada et al. [12], and Varma et al. [13], our case adds value by demonstrating the critical role of emergency-based correction before autoimmune confirmation, preventing fatal outcomes. Furthermore, the coexistence of hypothyroidism supports the autoimmune clustering often observed in SS [3,8]. The key learning point is that acute flaccid paralysis with hypokalemia should always prompt acid-base evaluation. Identifying a non-anion gap metabolic acidosis with inappropriately alkaline urine indicates dRTA, warranting autoimmune screening for SS even in the absence of sicca symptoms [8,9].

## Conclusions

Hypokalemic paralysis can rarely serve as the initial and sole presenting feature of SS, secondary to dRTA. Rapid identification and correction of potassium and acid-base imbalance in the emergency department are lifesaving. Subsequent autoimmune workup is essential for definitive diagnosis and long-term management. Early recognition and correction of electrolyte imbalance lead to complete neurological recovery and may delay long-term complications.
